# Electroconvulsive Therapy for Life‐Threatening Refractory Hypoactive Delirium in an 87‐Year‐Old Patient: A Case Report

**DOI:** 10.1155/crps/9267185

**Published:** 2026-10-30

**Authors:** Henri Perrin, Joao de Sousa, Patrizia d’Amelio, Pierre Vandel, Armin von Gunten, Kevin Swierkosz-Lenart, Beatriz Pozuelo Moyano

**Affiliations:** ^1^ Service of Old-Age Psychiatry Department of Psychiatry Lausanne University Hospital (CHUV) University of Lausanne Prilly Switzerland unil.ch; ^2^ Geriatrics Unit Department of Internal Medicine Lausanne University Hospital (CHUV) University of Lausanne Lausanne Switzerland unil.ch

**Keywords:** electroconvulsive therapy, persistent delirium, refractory delirium

## Abstract

Delirium is a frequent and serious neuropsychiatric syndrome in older inpatients and is associated with severe negative outcomes. Hypoactive delirium is often underdiagnosed and may become life‐threatening when accompanied by sustained refusal of oral intake. Evidence for pharmacological treatment of persistent or refractory delirium remains limited, and the role of electroconvulsive therapy (ECT) is poorly defined. We report an 87‐year‐old woman with both schizoaffective and major neurocognitive disorder who developed refractory hypoactive delirium in the context of multiple medical precipitants. Despite systematic identification and treatment of precipitants, correction of metabolic disturbances, and optimization of psychotropic medication, she continued to exhibit marked psychomotor inhibition and refusal of food and fluids, leading to clinically significant dehydration and electrolyte abnormalities. Given imminent medical risk and refractoriness to standard management, ECT was initiated twice weekly after multidisciplinary evaluation. Clinical improvement was observed after the first session, with resolution of delirium features and restoration of oral intake after three sessions, without periprocedural complications. This case suggests that carefully monitored ECT may be considered as a rescue intervention in cases of life‐threatening refractory hypoactive delirium and underscores the need for clearer escalation guidance and higher‐quality evidence.

## 1. Introduction

Delirium is a common and serious neuropsychiatric syndrome associated with major adverse outcomes. Clinically, delirium is commonly classified into hyperactive, hypoactive, or mixed subtypes based on the predominant pattern of psychomotor activity [1, 2]. Hypoactive delirium may be especially challenging to recognize and can progress to severe medical complications when associated with refusal of oral intake and care. To date, no pharmacological treatment has demonstrated consistent efficacy, and management relies primarily on identification and treatment of precipitating factors and on nonpharmacological multicomponent strategies [3]. Electroconvulsive therapy (ECT) is an established treatment for multiple psychiatric conditions, such as resistant mood disorders, schizophrenia, catatonia, and Behavioural and Psychological Symptoms of Dementia(BPSD), but its role in delirium remains poorly defined [4]. The only systematic review on this topic reported improvement with ECT, but the overall low quality of the evidence did not support ECT as a standard treatment for delirium [5]. This case report adds to the limited literature by describing the successful use of ECT in a patient with severe, life‐threatening hypoactive delirium.

## 2. Case Report

An 87‐year‐old woman with schizoaffective disorder and major neurocognitive disorder (baseline characteristics summarized in Table 1) was referred from her nursing home to the emergency department following the sudden onset of decreased alertness and gait instability. After comprehensive evaluation, hypoactive delirium was diagnosed, with a urinary tract infection (UTI) identified as a precipitating factor and successfully treated with cotrimoxazole. Symptoms persisted, with ongoing hypoactive delirium and poor oral intake. She was referred again to the emergency department and subsequently required admission on an internal medicine ward, where additional medical precipitants were identified and successfully treated, including acute urinary retention, constipation, and Candida oesophagitis. She was readmitted 3 days after discharge following an accidental overdose of quetiapine and lithium (311 ng/mL and 1.5 mmol/L, respectively), with worsening alertness, and psychogeriatric inpatient management was recommended.

**Table 1:** Baseline characteristics.

Comorbidities	
Psychiatric	Physical
• Schizoaffective disorder (6A21.2 ICD‐11)	• Atrioventricular (AV) block with postsinus pause/asystole; dual‐chamber permanent pacemaker implanted in 2019
• Major neurocognitive disorder, probable Alzheimer’s disease (6D80 ICD‐11)	

**Treatments**	

• Lithiofor 990 mg/day	• Macrogol 667 mg/mL 20 mL 3x/day
• Quetiapine XR 600 mg/day	
• Seresta 45 mg/day	

During her psychogeriatric hospitalization, hypoactive delirium was confirmed by clinical assessment according to DSM‐5 and to confusion assessment method (CAM) criteria, a widely used bedside diagnostic instrument for delirium (Table S2) [2, 6]. Catatonia was also considered in view of the schizoaffective comorbidity and the presence of stupor, low verbal production, and withdrawal; the Bush–Francis Catatonia Rating Scale (BFCRS), a clinician‐rated scale used to screen for and quantify catatonic signs, was quantified at 3 points (+1 stupor, +2 withdrawal, Table S1) [7]. Catatonia was not retained due to absence of catatonia‐defining positive signs (e.g., echophenomena, abnormal or automatic motor behaviors) and presence of cognitive fluctuations. An adjustment disorder to the intoxication episode, with severe withdrawal, was also considered but not retained given the appearance of symptoms well before the intoxication episode. Novel uriculture identified an *Enterococcus faecalis* infection, treated ineffectively with nitrofurantoin for 7 days, with persistent bacteriuria on repeat culture. Quetiapine was tapered and discontinued, aripiprazole introduced and titrated to 10 mg/d, and oxazepam stopped due to concern as to confusional worsening. Despite these measures, she continued to refuse oral intake and hydration. Delirium persisted for 35 days despite correction of infectious, metabolic, and retention‐related precipitants, discontinuation of sedating agents, and implementation of multicomponent nonpharmacological strategies.

Given the risks related to severe dehydration and malnutrition and the practical difficulty of providing adequate parenteral hydration because of poor treatment adherence, urgent ECT was considered as a rescue intervention for severe hypoactive delirium resistant to treatment of precipitating factors. This case involved factors associated with ECT increased procedural and neuropsychiatric risk, including major neurocognitive disorder, conduction disease with a permanent pacemaker, recent lithium/quetiapine intoxication, and electrolyte instability. These risks were evaluated and mitigated through multidisciplinary management: cardiology and anesthesia consultations prior to initiation, device‐aware periprocedural planning, intravenous correction of dehydration and electrolyte abnormalities (Na + 153 mmol/L, K + 2.7 mmol/L) with 1000 mL of 5% glucose solution, 50 mEq of KCl, and 2 g of magnesium sulfate and physiological and electrocardiographic monitoring. Residual nonmodifiable risks were deemed acceptable after individualized risk–benefit assessment in light of clinical urgency, and with written consent by her healthcare proxy, ECT treatment was initiated. No periprocedural complications occurred. Bilateral ECT was delivered twice weekly using a Mecta device. This method was chosen as the preferred approach based on the existing literature, which reports a greater number of cases and favorable outcomes with bilateral electrode placement compared to unilateral ECT in the management of delirium [5]. At the first session, seizure threshold was 128 mC (pulse width 0.5 ms, frequency 20 Hz, stimulus duration 8 s, and intensity 800 mA). Anesthesia was induced with etomidate and succinylcholine. Subsequent sessions used charges up to 384 mC (Figure 1). Clinical improvement was observed after the first session, with complete resolution of delirium features after three sessions, including progressive quantifiable restoration of oral intake and normalization of psychomotor activity (Figure 1). At discharge, she showed spontaneous discourse and adequate intake, with persistence of baseline disorientation and memory impairment. Given the recurrent nature of the patient’s delirium episodes, her ongoing vulnerability to precipitating factors, the rapid response to ECT, and the good tolerability of treatment, once‐weekly continuation ECT was initiated after resolution of the acute episode as an individualized, empirical relapse‐prevention strategy. This approach was also supported by literature suggesting a substantial risk of relapse of neuropsychiatric symptoms in patients with dementia following ECT discontinuation, although no evidence specifically addresses relapse in delirium [8]. A table of key events is provided in Figure 1. This case report was prepared in accordance with the CARE guidelines [9].

**Figure 1:**
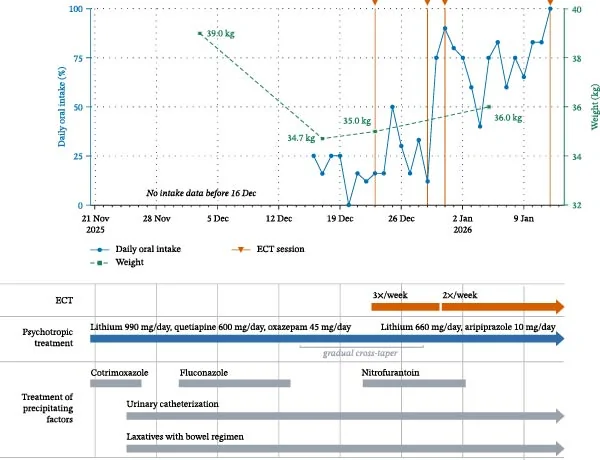
Timeline of key events.

## 3. Discussion

This case contributes to the limited literature describing ECT as a potential rescue intervention for severe, refractory delirium, particularly when delirium becomes medically life‐threatening due to persistent refusal of intake and failure of standard management [5]. In the present case, delirium was repeatedly confirmed across settings, and recovery occurred shortly after ECT initiation after a prolonged course with no meaningful improvement despite systematic precipitant management and psychotropic adjustments.

Importantly, ECT is a well‐established procedure with a long history of use across age groups, including older adults. ECT is generally regarded as a safe intervention even in medically complex geriatric patients, although cognitive adverse effects and periprocedural complications may be more frequent in the presence of frailty and pre‐existing neurocognitive disorder [10]. In our case, safety considerations (major neurocognitive disorder, conduction disease with permanent pacemaker, recent psychotropic intoxication, and electrolyte instability) were addressed through multidisciplinary evaluation, correction of reversible medical issues, and close physiological and electrocardiographic monitoring. Consistent with clinical practice, ECT should not be framed as having “absolute contraindications” in the strict sense; rather, candidacy is determined by an individualized risk–benefit assessment that considers urgency, reversibility of precipitants, available alternatives, and the feasibility of supportive care. In the present case, residual nonmodifiable risks were weighed against the immediate threat posed by ongoing refusal of intake and medical deterioration and were judged acceptable in light of the clinical urgency and the lack of effective alternative interventions [11]. Off‐label use of ECT for refractory delirium remains a gray area in international guidance, despite delirium being associated to a significant increase in mortality. While prevention and standard management are well described, high‐quality evidence addressing persistent or refractory delirium is scarce. Management therefore focuses on identifying and correcting precipitants and applying multicomponent nonpharmacological strategies [4], whereas pharmacological options remain largely symptomatic with limited supporting evidence [3]. When delirium persists despite appropriate medical and environmental management and becomes life‐threatening, clinicians have limited evidence‐based guidance for escalation. In our case, we considered delirium as refractory given the absence of improvement after several weeks despite correction of precipitant factors, discontinuation of sedating agents, psychotropic optimization, and implementation of multicomponent nonpharmacological strategies. ECT was considered as a rescue intervention when the condition became life‐threatening because of persistent refusal of oral intake and clinically significant dehydration and electrolyte abnormalities. Other potential interventions such as further antipsychotic trials were considered but not retained given the low scientific support and slow effect, while parenteral hydration or nasogastric tube feeding were not retained given poor treatment adherence and because they would not have addressed the underlying neuropsychiatric syndrome driving the persistent refusal of intake.

This case underscores the need for pragmatic recommendations on how to define refractoriness, when to escalate, and which outcomes to monitor. The relationship between ECT and delirium is also bidirectional. Delirium may occur as an adverse neuropsychiatric outcome in the peri‐ECT period, particularly in older adults with major neurocognitive disorder and concomitant psychotropic medications [12]. Conversely, ECT may improve delirium in selected cases, though mechanisms remain incompletely understood. This duality reinforces the importance of cautious selection, medical optimization, close monitoring, and conservative interpretation of apparent treatment effects. Several limitations constrain causal inference. First, diagnosis in this clinical context is inherently questionable, and given the lack of objective diagnostic tools, important differential diagnoses cannot be excluded with complete certainty. In particular, hypoactive delirium can overlap phenomenologically with catatonia and with severe mood episodes featuring marked psychomotor inhibition. Some authors conceptualize neuropsychiatric continuum, which may partially explain responsiveness to ECT. Catatonia was considered but not retained based on the low BFCRS score, absence of catatonia‐defining positive signs, lack of prior catatonic episodes, sudden onset and fluctuating symptoms, and presence of several prior episodes of hypoactive delirium. Recent work has shown that several symptoms counted as catatonia are nonspecific and also common in other psychiatric conditions, particularly hypoactive delirium. This suggests that, in the presence of delirium, applying the BFCRS may lead to overdiagnosis of catatonia. Indeed, in our case, the absence of key symptoms such as staring or unusual motor signs makes a diagnosis of catatonia inconsistent with the BFCRS scoring [13]. Key clinical features distinguishing catatonia from delirium are summarized in Table S3. Second, because multiple treatment interventions were administered concurrently, the independent contribution of ECT to the patient’s improvement cannot be determined, and a clear causality cannot be established. Nevertheless, the last UTI was ineffectively treated, and antipsychotics are not an etiological treatment for delirium, suggesting that ECT contributed substantially to patient’s improvement. Importantly, patient’s symptoms were present well before the onset of electrolyte imbalances, which were actually consequences of patient’s condition. Therefore, their correction is unlikely to be the cause of delirium’s resolution. Finally, follow‐up interpretation is limited by continuation of maintenance ECT after discharge, and durability of recovery after full discontinuation cannot be determined. Future reports and studies would benefit from clearly documented outcomes after cessation of ECT, including relapse monitoring and longer‐term functional endpoints. Ultimately, higher‐quality evidence is needed. Prospective observational cohorts and pragmatic trials, focusing specifically on refractory delirium and using standardized diagnostic and outcome measures, would be necessary to clarify which patients may benefit, define safety profiles in older medically complex populations, and distinguish delirium improvement attributable to ECT from recovery driven by precipitant resolution.

## 4. Conclusions

This report illustrates a case of refractory hypoactive delirium associated with life‐threatening refusal of oral intake with metabolic complications. In a medically complex patient with failure to improve despite optimization of precipitating factors and pharmacological adjustments, urgent modified ECT was temporally associated with rapid clinical recovery and restoration of oral intake. Although causality cannot be established from a single case and important confounders remain, this report supports careful consideration of ECT as a multidisciplinary, carefully monitored rescue option in selected cases of severe refractory delirium and underscores the need for higher‐quality prospective research to define indications, safety, and outcomes.

## Funding

Henri Perrin was supported by the CHUV Department of Medicine (DM) and Department of Laboratory Medicine and Pathology (DMLP) Research Grant 2025 (Lausanne University Hospital, CHUV). Beatriz Pozuelo Moyano and Kevin Swierkosz‐Lenart have received funding from the research grant from the Department of Psychiatry (Lausanne University Hospital). Beatriz Pozuelo Moyano has received funding from the Tremplin Grant (University of Lausanne) and research grant for medical scientists (Faculty of Biology and Medicine, University of Lausanne) for academic development and protected research time.

## Ethics Statement

As a single‐patient report of routine clinical care with no research intervention, approval from the Ethics Committee was not required. The report follows the Declaration of Helsinki and CARE guidelines [9].

## Consent

As the patient lacked decision‐making capacity, written informed consent for publication of this deidentified report was obtained from her legally authorized healthcare proxy. A copy of the written consent is available for review by the journal’s Editor‐in‐Chief.

## Conflicts of Interest

The authors declare no conflicts of interest.

## Supporting Information

Additional supporting information can be found online in the Supporting Information section.

## Supporting information


**Supporting Information** Table S1: Bush‐Francis Catatonia Rating Scale (BFCRS). Table S2: Confusion Assessment Method (CAM). Table S3: Clinical features distinguishing catatonia from delirium.

## Data Availability

The data that support the findings of this study are available from the corresponding author upon reasonable request.
