# Supplementary material for: Electroconvulsive Therapy for Life‐Threatening Refractory Hypoactive Delirium in an 87‐Year‐Old Patient: A Case Report
Source: Case Rep Psychiatry. 2026 Oct 30;2026:9267185. doi: 10.1155/crps/9267185 (PMC13721894; doi:10.1155/crps/9267185)
Supplement: Supplementary file 1 [file CRPS-2026-9267185-s001.docx]

**BFCRS vs. CAM — Clinical Assessment & Differential Diagnosis**

| **BUSH-FRANCIS CATATONIA RATING SCALE (BFCRS)** | | | | | | |
| --- | --- | --- | --- | --- | --- | --- |
| **#** | **Item** | **Anchor (0 → 1 → 2 → 3)** | **0** | **1** | **2** | **3** |
| ***MOTOR / BEHAVIORAL*** | | | | | | |
| **1** | Excitement | *Mild restlessness → frantic, needs restraint* | ☐ | ☐ | ☐ | ☐ |
| **2** | Immobility / Stupor | *Slowed → motionless, no response to pain* | ☐ | ☐ | ☐ | ☐ |
| **3** | Mutism | *Whispers → monosyllabic → no speech* | ☐ | ☐ | ☐ | ☐ |
| **4** | Staring | *Poor eye contact → fixed gaze >30 s* | ☐ | ☐ | ☐ | ☐ |
| **5** | Posturing / Catalepsy | *Held <1 min → 1–10 min → >60 min* | ☐ | ☐ | ☐ | ☐ |
| **6** | Grimacing | *Occasional → frequent → constant* | ☐ | ☐ | ☐ | ☐ |
| **7** | Echopraxia / Echolalia | *Occasional → frequent → constant mimicking* | ☐ | ☐ | ☐ | ☐ |
| **8** | Stereotypy | *Occasional → constant, non-interruptible* | ☐ | ☐ | ☐ | ☐ |
| **9** | Mannerisms | *Occasional → constant odd purposeful movements* | ☐ | ☐ | ☐ | ☐ |
| **10** | Verbigeration | *Occasional → nearly only speech produced* | ☐ | ☐ | ☐ | ☐ |
| ***NEUROLOGICAL / TONE*** | | | | | | |
| **11** | Rigidity | *Mild → moderate → cannot be repositioned* | ☐ | ☐ | ☐ | ☐ |
| **12** | Negativism | *Passive → active → forceful resistance* | ☐ | ☐ | ☐ | ☐ |
| **13** | Waxy Flexibility | *Slight → sustained waxy resistance* | ☐ | ☐ | ☐ | ☐ |
| **14** | Withdrawal | *Eats if urged → refuses food → tube feeding* | ☐ | ☐ | ☐ | ☐ |
| **15** | Impulsivity | *Occasional → frequent → unpredictable* | ☐ | ☐ | ☐ | ☐ |
| **16** | Automatic Obedience | *Mild → moderate → total compliance* | ☐ | ☐ | ☐ | ☐ |
| **17** | Mitgehen | *Mild arm raise → occurs with minimal touch* | ☐ | ☐ | ☐ | ☐ |
| **18** | Gegenhalten | *Mild → full resistance proportional to force* | ☐ | ☐ | ☐ | ☐ |
| **19** | Ambitendency | *Occasional → constant motor indecision* | ☐ | ☐ | ☐ | ☐ |
| **20** | Grasp Reflex | *Unilateral → bilateral despite instruction* | ☐ | ☐ | ☐ | ☐ |
| **21** | Perseveration | *Mild → constant, unredirectable* | ☐ | ☐ | ☐ | ☐ |
| **22** | Combativeness | *Mild → severe, needs restraint* | ☐ | ☐ | ☐ | ☐ |
| **23** | Autonomic Instability | *Mild T°/HR/BP changes → severe/NMS-like* | ☐ | ☐ | ☐ | ☐ |
| **TOTAL (diagnosis: ≥2 items ≥1 OR total ≥3)** | | | **_______3 / 69** | | | |

**Supplementary Table 1 :** Bush-Francis Catatonia Rating Scale (BFCRS).

| **CONFUSION ASSESSMENT METHOD (CAM)** | | | |
| --- | --- | --- | --- |
| **Feature** | **Clinical Question** | **Present?** | **Observations / Notes** |
| ***REQUIRED (both must be present)*** | | | |
| **1. Acute onset & fluctuating course** | Acute change from baseline? Does it fluctuate during the day? | ☐ Yes ☐ No |  |
| **2. Inattention** | Difficulty focusing? Easily distracted? (digit span, serial 7s, months backwards) | ☐ Yes ☐ No |  |
| ***PLUS at least ONE of:*** | | | |
| **3. Disorganized thinking** | Rambling, incoherent, or unpredictably switching topics? | ☐ Yes ☐ No |  |
| **4. Altered consciousness** | Not alert: vigilant / lethargic / stuporous / comatose? | ☐ Yes ☐ No |  |
| **CAM POSITIVE IF:** | | **Features 1 + 2 AND (3 or 4) ☐ YES ☐ NO** | |

**Supplementary Table 2 :** Confusion Assessment Method (CAM).

| **DIFFERENTIAL DIAGNOSIS SUMMARY** | | |
| --- | --- | --- |
| **Feature** | **Catatonia (BFCRS +)** | **Delirium (CAM +)** |
| **Onset** | Variable (acute or insidious) | Acute (hours–days) |
| **Course** | Sustained, less fluctuating | Fluctuating throughout the day |
| **Motor signs** | Specific: posturing, rigidity, waxy flexibility, stereotypies | Non-specific agitation or retardation |
| **Inattention** | May appear inattentive (mutism/stupor) | Core required feature |
| **Consciousness** | Usually preserved | Frequently clouded |
| **Hallucinations** | Less common | Common (especially visual) |
| **Lorazepam challenge** | Dramatic improvement | Not responsive; may worsen |
| **EEG** | Normal or mild slowing | Diffuse slowing (characteristic) |
| **Co-existence?** | YES — 'Catatonic Delirium' recognized; both scales may be simultaneously positive |  |

*Bush et al., Acta Psychiatr Scand 1996 | Inouye et al., Ann Intern Med 1990*

**Supplementary Table 3.** Clinical features distinguishing catatonia from delirium.
